# Confinement primes cells for faster migration by polarizing active mitochondria†

**DOI:** 10.1039/d3na00478c

**Published:** 2023-11-22

**Authors:** Jenna A. Mosier, Emily D. Fabiano, Catherine M. Ludolph, Addison E. White, Cynthia A. Reinhart-King

**Affiliations:** a Department of Biomedical Engineering, Vanderbilt University Nashville TN USA cynthia.reinhart-king@vanderbilt.edu; b Department of Chemical Engineering, University of Texas at Austin Austin TX USA

## Abstract

Mechanical cues in the tumor microenvironment interplay with internal cellular processes to control cancer cell migration. Microscale pores present in tumor tissue confer varying degrees of confinement on migrating cells, increasing matrix contact and inducing cytoskeletal rearrangement. Previously, we observed that increased collagen matrix contact significantly increased cell migration speed and cell-induced strains within the matrix. However, the effects of this confinement on future cell migration are not fully understood. Here, we use a collagen microtrack platform to determine the effect of confinement on priming MDA-MB-231 cancer cells for fast migration. We show that migration through a confined track results in increased speed and accumulation of migratory machinery, including actin and active mitochondria, in the front of migrating breast cancer cells. By designing microtracks that allow cells to first navigate a region of high confinement, then a region of low confinement, we assessed whether migration in high confinement changes future migratory behavior. Indeed, cells maintain their speed attained in high confinement even after exiting to a region of low confinement, indicating that cells maintain memory of previous matrix cues to fuel fast migration. Active mitochondria maintain their location at the front of the cell even after cells leave high confinement. Furthermore, knocking out vinculin to disrupt focal adhesions disrupts active mitochondrial localization and disrupts the fast migration seen upon release from confinement. Together, these data suggest that active mitochondrial localization in confinement may facilitate fast migration post-confinement. By better understanding how confinement contributes to future cancer cell migration, we can identify potential therapeutic targets to inhibit breast cancer metastasis.

## Abstract 2

Priming in confined, collagen microtracks increases speed and mitochondrial localization to the front of the cell, even after cells have transitioned out of confinement. Disrupting proper focal adhesion formation decreases this memory.

## Graphical abstract

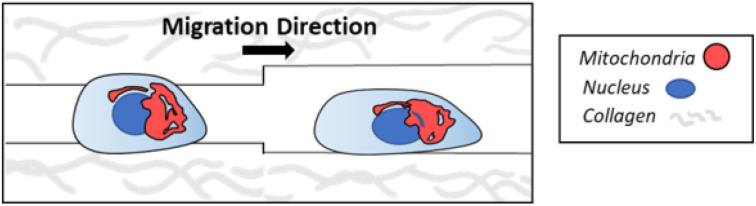

## Introduction

Breast cancer tissue is characterized by a highly heterogeneous extracellular matrix (ECM) architecture.^1^ Cancer, stromal, and immune cells receive and respond to mechanical cues in the microenvironment, adapting and subsequently changing their behaviors, a process known as mechanotransduction.^2^ When cells adhere to the surrounding ECM, composed primarily of collagen type I, cell surface integrins coordinate mechanical signals to modify cellular migratory ability.^3,4^ In metastasis, cancer cells use this ability to navigate the dense and disordered topography and physical properties of the primary tumor microenvironment to maneuver through the tissue towards the lymphatic system or bloodstream to spread throughout the body.^5^ During this migration, cells encounter disparately sized pores and tunnels that can constrict the cell body, increase matrix contact, and impede typical migration.^6^ When faced with such obstacles, cells are required to either degrade the surrounding ECM or deform their shape to enter and migrate through these spaces.^7^ Cells rely on dynamic phenotypic switching to adapt to the surrounding microenvironment during migration. While typical mesenchymal migration relies on adhesion-based cycling of leading-edge protrusion and rear-contraction, in highly confined, PDMS microchannels, cells have been shown to adopt an amoeboid phenotype that utilizes fewer adhesions and bleb-based motility to squeeze through tight spaces.^1,8–11^ Several methods for confined migration have been observed, including the ion gradient-driven osmotic engine model^12,13^ and the pressure-driven nuclear piston model.^14^ It has recently been shown that the polarization of key plasma membrane proteins, NHE1 and SWELL1, regulates isotonic swelling of MDA-MB-231 cells to regulate and increase the efficiency on confined migration.^13^ The role of spatial confinement on cell behavior and migratory ability has previously been studied using microfabricated tools and hydrogels mimicking the pore geometries found *in vivo*, typically created from polydimethylsiloxane (PDMS), a supraphysiologically stiff environment for cells.^12,15,16^ In softer, more physiologically relevant microtracks fabricated completely from collagen,^17^ we have shown that fully confined cancer cells utilize an intermediate migratory mode between mesenchymal and amoeboid, in which cells still exhibit focal adhesions, though fewer than cells not fully confined, but have increased migration speed and a rounder morphology.^17,18^ However, the effects of confinement on future cell migration have not been fully explored.

Cell metabolism has received increasing attention as a regulator of confined cell migration.^19–22^ Previously, we showed that cells increase both ATP : ADP and glucose uptake in confined collagen microtracks to fuel fast migration.^23^ Additionally, intracellular ATP : ADP ratios and their spatial gradients within the cell body are intrinsically linked to directionality and velocity during migration.^23–25^ Spatial localization of energy production and utilization drives protrusion formation and actin remodeling necessary for migration.^26^ Mitochondria, key contributors to energy production and regulation, are moved throughout the cell body on microtubules and the actin cytoskeleton,^27,28^ likely to fuel key processes in different regions of the cell. Previously, anterior mitochondrial localization in confined cells has been associated with higher migration speeds, persistence, and protrusion formation, while inhibition of that connection through knockout of the Rho GTPase Miro1, a linker between mitochondria and microtubules, decreases migration speed.^25,29^ Mechanical jamming and unjamming in epithelial monolayers, a form of confinement, also affect cell redox ratios, NADH, and glucose uptake,^30^ indicating that spatial constriction induces distinct metabolic changes in cellular bioenergetics.

Cells migrating in high degrees of confinement adopt a less mesenchymal, more amoeboid migratory mode, characterized by faster migration and fewer focal adhesions.^8,9^ Interestingly, depletion of vinculin, a key focal adhesion protein that connects the ECM to the cytoskeleton, disrupts unidirectional cell migration in soft, collagen microtracks.^31,32^ This suggests that while not critical for motility, vinculin contributes to how cells navigate confined spaces, likely due to the force-stabilization vinculin provides to focal adhesions.^33^ Vinculin is not only force sensitive, but also serves as a mechanical linker between the actin cytoskeleton and focal adhesions, and therefore, plays a critical role in mechanotransduction cascades. While focal adhesion formation and dynamics have been previously studied in confined migration, the specific role that vinculin plays in confined migration and the ramifications its depletion may have on the ability of cells to navigate confined spaces is not well understood.

Priming in stiffer mechanical environments has previously been shown to induce mechanical memory in cells, such that they retain high migration velocity induced by high stiffness even after the stimulus is gone.^34^ Mammary epithelial cells, fibroblasts, and mesenchymal stem cells have all been reported to maintain properties and behaviors, such as migration, contractility, or differentiation, incurred through culture on stiff substrates even after being transferred to softer surfaces, indicating that stiffness-dependent memory is a property of many different cell types.^34–36^ Recent work has shown that metastatic breast cancer cells can be primed in elevated fluid viscosities to promote an increase in migration and metastatic ability.^37^ While cancer cell migration in confined microenvironments has been studied, the effects of confinement on future migration are not well understood. Here, we use microfabricated tunnels in collagen to show that spatial constriction increases migration speed and induces redistribution of mitochondria to the cell front. Mitochondria and actin remains polarized at the front of the cell, correlating with fast migration even after the cells leave confinement. Knockout of vinculin indicates that while vinculin is nonessential for fast migration in confinement, it contributes to priming of cells in confinement to polarize mitochondria and promote memory during future migration.

## Materials and methods

### Cell culture and reagents

Highly metastatic MDA-MB-231 female human mammary adenocarcinoma cells (HTB-26, ATCC, Manassas, VA) and 4T1 murine cells (ATCC) were maintained at 37 °C and 5% CO_2_ in Dulbecco's Modified Eagle's Medium (DMEM; Thermofisher) supplemented with 10% fetal bovine serum (FBS; Atlanta Biologicals) and 1% penicillin/streptomycin (Thermofisher). MDA-MB-231 were stably transduced with Life-Act eGFP (#84383, Addgene, Watertown, MA).

### Microtrack fabrication

Collagen microtracks were prepared as previously described.^17^ All microfabrication processes were carried out in the Vanderbilt Institute of Nanoscale Science and Engineering (VINSE) cleanroom. Briefly, a darkfield, chrome-coated glass mask was printed with microtrack geometries designed in K-Layout and transferred to the mask using a Heidlberg μPG 101. Photolithography techniques were then used to coat a 100 mm diameter silicon wafer with either S1813 or SU8 photoresist that was then exposed with light with an energy density of 82 mJ cm^−2^ or 120 mJ cm^−2^, respectively. The wafer patterned with S1813 resist then underwent a Bosch etch process using an Oxford etcher for 31 cycles to achieve the desired depth and fluorinated using trichloro-perfluor-octylsilane. To achieve a correct aspect ratio for temporarily confined microtracks on the wafer patterned with SU8 resist, the wafer was then developed, cleaned, and fluorinated using trichloro-perfluor-octylsilane to prevent polymer adherence. Wafers were then used as template molds to create stamps. Wafers were cast in polydimethylsiloxane (PDMS; Dow Corning) crosslinker and monomer at a 1 : 10 ratio and cured at 60 °C for at least 4 h. Stamps containing various microtrack patterns were then cut from the PDMS mold and used for collagen micromolding.

Rat tail tendon type I collagen stock solution (10 mg mL^−1^, prepared in-house) was diluted to 3 mg mL^−1^ with complete culture medium and neutralized with 1 N NaOH. PDMS stamps were briefly coated in diluted collagen solution, and, after excess collagen was aspirated, inverted over 150 μL of collagen solution placed between two plastic spacer strips. The collagen solution and stamp set-up was allowed to polymerize for at least 90 min at 37 °C. The PDMS stamps were gently pulled away from the collagen, resulting in completely collagen tracks of varying widths (ESI Fig. 1A†). Cells were seeded dropwise on top of the collagen between the spacers at a low density of 100 000 cells per mL to attempt to seed single cells per track. The entire system was then covered with a collagen-coated glass coverslip, sealing the microtracks. Fresh media supplemented with or without treatment as indicated was added to microtracks after allowing the polymerization of the lids to the microtracks. The media was supplemented with either the vehicle control, DMSO (Sigma, D8414), or 500 μM antimycin-A (AMA; Millipore Sigma, A8674), 75 nM Mitotracker Green (Invitrogen, M7514) or tetramethylrhodamine, methyl ester (TMRM; Invitrogen, U34361) for mitochondrial localization assays. The final length of the collagen microtracks was 1000 μm, with a uniform height 15 μm as previously described.^18^ Straight, uniformly confined and uniformly unconfined microtracks were created with widths of 7 and 15 μm, respectively, in addition to microtracks that alternate every 75 μm from 7 to 15 μm in width. Additionally, temporarily alternating microtracks consisted of an alternating 7 to 15 μm region before emptying into a continuous, 15 μm in width track and temporarily confined microtracks consisted of a continuous, uniformly confined (7 μm) region that entered into a continuously unconfined (15 μm) region. The collagen microtracks were prepared on plastic bottom 6-well plates for phase-contrast imaging and glass bottom 6-well plates with no. 1.5 cover glass (Cellvis) for confocal imaging. After seeding with cells and supplementing fresh media, the entire system was placed in an incubator for 5 h to allow for cell spreading, and then cells were placed in microscope environmental chamber to acclimate to temperature and imaging was performed (ESI Fig. 1B†).

### Phase and confocal microscopy

Time-lapse phase contrast imaging was performed on a Zeiss Axio Observer Z1 inverted microscope equipped with a Hamamatsu ORCA-ER camera using a 10×/0.3 NA objective and operated by AxioVision software. For cell speed measurements, MDA-MB-231 cells were imaged every 20 min for at least 12 h, beginning ∼6 h after seeding to allow for cell spreading. For counting reversals in MDA-MB-231 and VclKO cells, imaging was performed every 10 min to capture changes. To measure mitochondrial localization signal, time-lapse confocal imaging was performed every 20 min for at least 12 h using a 20×/0.8 NA objective. All live-cell imaging was performed in an environmental chamber maintained at 37 °C and 5% CO_2_.

### Migration analysis

Cell migration speed in collagen microtracks was calculated by measuring the distance between cell centroids (from frame to frame in the time-lapse series) and dividing by the total time interval. Cell centroid position was determined by manually outlining cells in Fiji ImageJ software.^38^ Speed measurements were taken over a minimum of 6 h. Outlines of cell area in each position in the microtrack during migration were analyzed using shape descriptors from Fiji ImageJ. Cells that divided or interacted with other cells during migration were excluded from analysis. In alternating width microtracks, cell migration speed was averaged over each region and compared to consecutive regions. In temporarily confined tracks, migration was calculated across the entire path, and post-analysis, cells were sorted into those that experienced <100, 100–200, 200–300, or >300 μm of confinement before transitioning were averaged.

Persistence was calculated as net distance traveled over 1 h divided by the total distance per 20 min intervals to achieve a value between 0 and 1, with 1 being most persistent. Reversals were determined by first calculating the displacement in the *X* direction according to the centroid between consecutive time points. The total distance traveled before a change in direction was observed was summed together for the duration of the migration video. Whenever the cell changed direction in the track, if the displacement of the centroid was greater than 10 μm, it was counted as a reversal. For each replicate, the number of cells experiencing 0, 1–2, 3–4, or 5+ reversals was calculated.

### Mitochondria and actin localization and mitochondrial activity

To determine mitochondrial or actin front-to-rear ratio in migrating cells, microtracks were fabricated as described and supplemented with TMRM to label active mitochondria in live MDA-MB-231 cells, or LifeAct-GFP cells were seeded into the tracks. The background was subtracted using the rolling-ball method. Cells were manually observed during each video to determine direction, and at the distances indicated in each figure, the line-scan function in Fiji was used to quantify the gradient of signal intensity (TMRM or LifeAct-GFP) across the cell body, from the leading edge (front) to the trailing edge (rear), normalized to the maximum intensity per cell. Cell length was divided in half, and the sum of signal in the front half was divided by the sum of signal in the rear half to achieve a front : rear ratio. In the instance of any cells changing direction during the frame of interest, the frames before and after frame of interest were used to determine new direction.

### Vinculin knockout cell line generation

This work was completed with the help of Emily D. Fabiano. MDA-MB-231 cells were transfected with CRISPR/Cas9 ribonucleoprotein (RNP) complexes consisting of S. pyogenes Cas9 pre-designed multi-guide RNAs targeting exon 1 (guide 1: GCCGCCUGCACGGCGGCCAC, guide 2: GAUAAUGCACGAGGAGGGCG, guide 3: AUCGUGCGCGUAUGAAACAC) to knockout vinculin (Gene KO Kit v2, Synthego) using a gene pulser (Bio-Rad Gene Pulser). Electroporation was performed at 120 V and 950 μF to deliver the RNP complexes to the cells. Following expansion of the transfected cells, single cells were seeded into wells of a 96 well-plate to perform a clonal dilution. Single cell clones were expanded, and western blotting was used to validate the vinculin knockout. Genomic DNA was extracted from these clones using the DNeasy Blood and Tissue Kit (Qiagen 69504). This genomic DNA was amplified using primers GAAAAGGGACCAGTAGGAGT and GCAGAAGTATTAGAAAGGAGGA and Sanger sequencing (Genewiz) was performed on the amplicons to determine editing efficiency using the online inference of CRISPR Edits (ICE) analysis tool from Synthego.

### Western blotting

Following CRISPR electroporation, lysates from vinculin knockout cells receiving the RNP complexes and not undergoing electroporation (0 V control), cells receiving only Cas9 and undergoing electroporation (Cas9 control), or the vinculin knockout colonies, were resolved by SDS-PAGE and transferred to polyvinylidene fluoride (PVDF) membranes. Total protein was stained using Revert 700 Total Protein Stain Kits for western blot Normalization (LI-COR 926-11010), according to the manufacturers protocol. 5% milk in TBS-Tween was used to block membranes for 1 h before incubating at 4 °C overnight with mouse anti-vinculin (Millipore V9131) (1 : 1000) or mouse anti-GAPDH (MAB374, Sigma) (1 : 1500). Membranes were incubated at room temperature for 1 h in 800 IRDye goat anti-mouse (LI-COR 431386) (1 : 5000) in 5% milk in TBS-Tween. Membranes were then imaged using an Odyssey Fc (LI-COR Biosciences, Lincoln, NE).

### Statistical analysis

Data in graphical form are presented as scatter plots, scatter plots with bars, reporting mean ± SEM, or *XY* plots showing mean ± SEM. Statistical analysis was conducted using GraphPad Prism 9.3. Normality in the spread of data for each experiment was tested using the D'Agostino–Pearson omnibus normality test. To evaluate statistical significance, analysis of variance (Kruskal–Wallis) with a Dunn's multiple comparisons test was used to compare more than two groups and two-tailed Mann–Whitney was used to compare two groups. For reversals, a two-way ANOVA with multiple comparisons using Šídák's multiple comparisons test was used. Statistical significance was considered with a *p*-value <0.05. Linear regression was compared using the Extra Sum of Squares F-Test to determine if slopes were statistically different from zero or significantly different from each other during comparison. All data are representative of a minimum of at least three independent replicate studies, with replicate number and sample size listed in each figure.

## Results

### Priming in confinement confers migratory speed increase in breast cancer cells

**Fig. 1:**
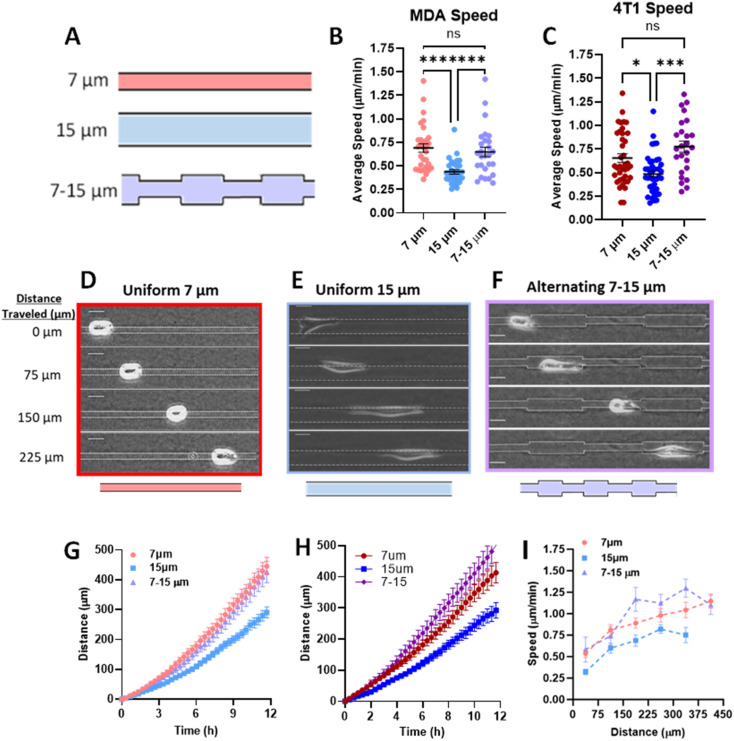
Priming in short confinement confers an increase in migration speed. (A) Schematic of microtrack patterns 7 μm (confined), 15 μm (unconfined), 7 – 15 μm (alternating width); (B) average speed of MDA-MB-231 cells in 7, 15, or 7–15 μm microtracks (*N* = 5–6, *n* = 27–31); (C) average speed of 4T1 cells in 7, 15, or 7–15 μm microtracks (*N* = 3–4, *n* = 23–41); (D–F) representative images of MDA-MB-231 cells in 7 μm, 15 μm, and alternating 7 to 15 μm microtracks, scale bar = 20 μm; (G) distance travelled by MDA-MB-231 cells in 7, 15, or 7–15 μm microtracks (*N* = 5–6, *n* = 27–31); (H) distance travelled by 4T1 cells in 7, 15, or 7–15 μm microtracks (*N* = 3–4, *n* = 23–41); (I) speed as a function of distance in 7, 15, or 7–15 μm microtracks (*n* = 12–16). * denotes *p*-value < 0.05, *** <0.001, **** <0.0001.

Previously, we have shown that cells in fully confined microtracks, in contact with all four surrounding walls of a channel, move significantly faster than cells that only reach a fraction of channel walls, adopting an intermediate migratory mode to efficiently navigate high degrees of confinement.^18^ To determine whether cells maintain that fast migration post-confinement, we fabricated 3D collagen microtracks with various confining geometries (Fig. 1A). Uniform microtracks of 15 μm height and 7 μm and 15 μm width were fabricated to represent confined and unconfined conditions, respectively. Confined regions were designed to be 7 μm based on a previous study in which it was determined that in a microtrack of decreasing width, cells were less likely to migrate into regions smaller than 7 μm,^23^ while unconfined regions were 15 μm in width. To interrogate short-term memory, microtracks that alternate from 7 μm to 15 μm in width were fabricated, with each region spanning 75 μm in length and maintaining a uniform height of 15 μm. In these tracks, termed ‘alternating width,’ cells repeatedly enter intermittent regions of confinement during microtrack migration. Agreeing with previous findings, the average speed of cells in confinement was significantly greater than that of cells in unconfined microtracks in both metastatic human breast cancer MDA-MB-231s and metastatic murine breast cancer 4T1s (Fig. 1B and C). Consistent with previous findings,^18^ cells in confinement tend to adopt a more rounded morphology as they interact with all four surrounding walls, while unconfined cells tend to be more elongated (Fig. 1D and E). Interestingly, cells in alternating width tend to switch between the two morphologies as they enter different regions of the track (Fig. 1F). The displacement of the centroid was measured for each timestep and used to calculate distance and speed. Cells in confinement travelled farther than cells that were unconfined (Fig. 1G and H). Interestingly, the average speed and distance travelled by cells in the alternating width microtracks matched that of confined cells and was significantly greater than unconfined cells (Fig. 1B–H). When comparing the speed of representative cells at each position in the microtrack, cells in alternating width tracks move at speeds similar to that of the confined cells (Fig. 1I), despite encountering intermittent unconfined regions. While a moderate speed increase was observed as cells entered the confined regions of the alternating width track, no significant difference between each region occurred. Overall, speed significantly increased as cells moved through the track. Even in the unconfined regions of the alternating track, a width at which cells would typically be expected to move at slower speed, cells maintain increased speed obtained while migrating through confined regions. Together, these results suggest that MDA-MB-231 and 4T1 cells exhibit short-term memory induced by repeated confinement that increases their speed.

**Fig. 2:**
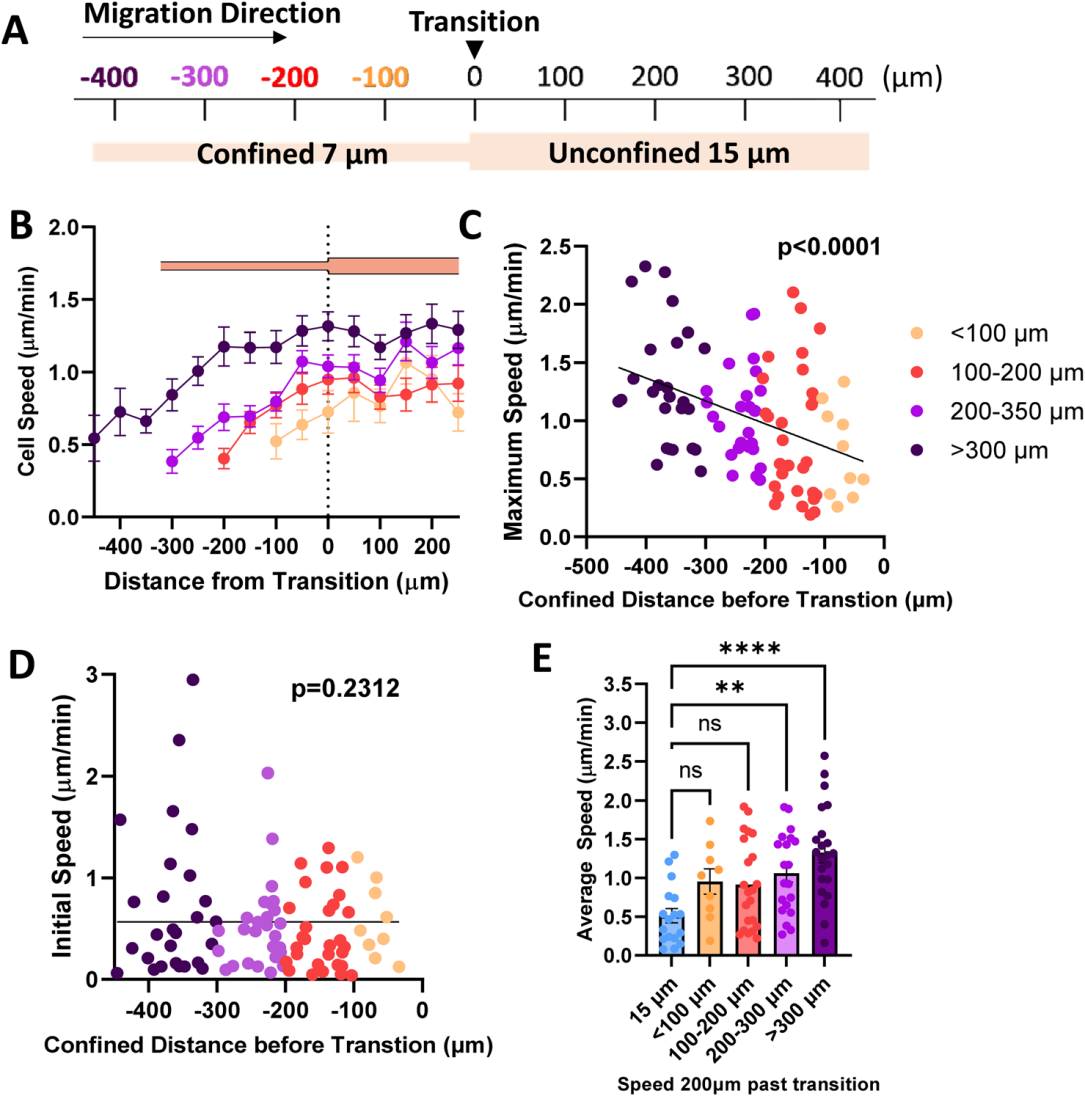
Priming in confinement results in memory of migration speed. (A) Schematic of temporarily confined track with cells sorted based on starting position from transition point; (B) speed as a function of position in the temporarily confined track (*N* = 26, *n* = 92, 13–27 cells per group); (C) maximum speed reached by cells in the confined portion of the track as a function of starting position where 0 denotes transition from 7 to 15 μm region of the temporarily confined track (*N* = 26, *n* = 92); (D) initial speed as a function of starting position (confined distance) in the 7/15 μm microtrack (*N* = 26, *n* = 92); (E) speed of cells 200 μm past the transition point, compared to cells in uniform 15 μm controls (*N* = 26, *n* = 73, 9–22). (C and D) Slopes tested using Extra Sum-of-Squares F-Test to determine if significantly different from 0. ** denotes *p*-value < 0.01, **** <0.0001. *P*-Values for best fit curves shown.

Since our data shows that cells maintain elevated speed even in alternating width microtracks with intermittent regions of unconfinement, we investigated whether priming in confinement can result in persistent migratory changes over longer distances. We fabricated microtracks containing a 400 μm region of confinement that transitions into a 400 μm unconfined region, termed ‘temporarily confined’. Cells were seeded into the temporarily confined track and allowed to navigate through each region. We then binned cells based on their initial distance from the transition point to the unconfined region (Fig. 2A). Cells increased speed in the confined portion of the track, and interestingly, upon exit migrated at the same rate as their maximum speed attained in the confined region of the track rather than decreasing in speed (Fig. 2B). The maximum speed cells reached in the confined region was found to be dependent on the distance of confinement, such that cells starting farther from the transition point attained higher maximum speeds by the time they reached the transition point (Fig. 2C). To confirm that we did not select for faster cells that were able to migrate the length of the temporarily confined track that could bias the data, initial cell speed and starting position were compared (Fig. 2D). Though a small fraction of the cells confined <300 μm initially start at higher speeds, there is not a significant relationship between starting position and cell speed as measured using an Extra Sum-of-Squares F-Test, indicating a relatively non-biased sample. To further explore how cell speed changed upon exit to the unconfined region, we compared their speed 200 μm past the exit to cells never having experienced any confinement. Cells confined greater than 200 μm before the transition exhibited speeds that were elevated at 200 μm past the exit compared to control unconfined cells (Fig. 2E), indicating that increased confinement may result in increased memory of MDA-MB-231 migration speed experienced in confinement.

### Confined microtracks induce polarized metabolic and migratory machinery

**Fig. 3:**
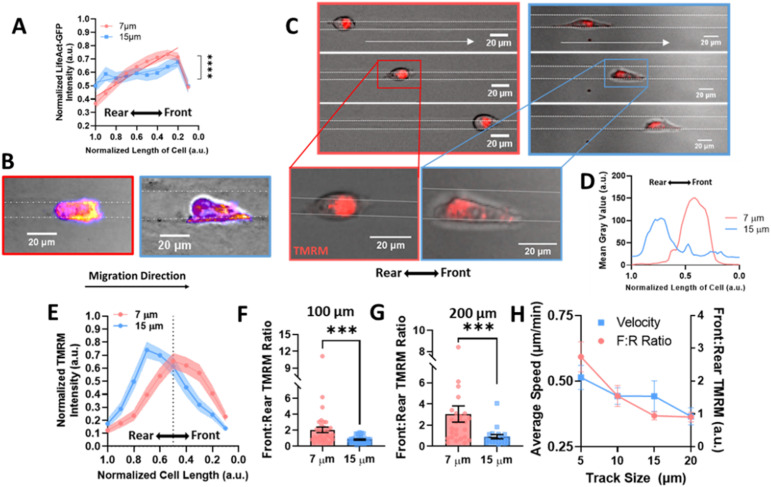
Actin and mitochondrial localization gradient increases at the front of the cell in confinement. (A) Gradient of actin fluorescent intensity across length of cell body where 0 is the front of the cell, normalized to 1 in 7 μm or 15 μm tracks (*N* = 4, *n* = 22/condition); (B) representative images of actin in cells, labelled with Fire LUT, scale bar = 20 μm; (C) representative images of cells labelled with TMRM in confined and unconfined tracks, moving right, scale bar = 20 μm; (D) line scan of a representative cell in a 7 or 15 μm track with TMRM, (E) gradient of TMRM signal across length of cell body where 0 is the front of the cell, normalized to 1 in 7 or 15 μm tracks (*N* = 5–6, *n* = 31–34); (F–G) front : rear TMRM signal in 7 and 15 μm tracks after migrating (F) 100 μm or (G) 200 μm in the tracks (*N* = 5–6, *n* = 31–34, 19–27); (H) average speed (left) or front : rear TMRM signal (right) in 5, 10, 15, or 20 μm tracks (*N* = 3, *n* = 26–32); *** denotes *p*-value < 0.001.

Migration requires the coordination of cytoskeletal components to drive polarization of the cell body, actin polymerization, organelle transport, and the formation of adhesions to facilitate forward motion.^39^ Since it has previously been observed that actin is increased in the leading portion of fast migrating cells,^40^ we looked to actin organization within the cell body in confined microtracks using stable expression of LifeAct-eGFP as a potential mechanism to explain how confinement induces a lasting effect on migration speed. Actin gradients were significantly steeper along the length of confined cells, with higher actin intensity measured at the leading edge of the cell (Fig. 3A, B and ESI Fig. 2A, B†), indicating that front-to-rear polarity of the cell cytoskeleton may confer a migratory advantage in confinement.

As mitochondria can be trafficked along actin in the cell body to regions of high energy demand^28,41,42^ and we see an increase in actin at the cell front in confinement, we investigated mitochondrial organization to determine if it was altered in confinement. Active mitochondria were labeled using TMRM and imaged in cells migrating in confined or unconfined microtracks (Fig. 3C and ESI Fig. 3A, B†). The gradient of active mitochondria across the length of the cell body was quantified using a line scan (Fig. 3D). Consistent with previous studies,^29^ active mitochondria were more localized to the front of the cell in confined microtracks compared to unconfined cells (Fig. 3E). Active mitochondrial content in the front half of the cell compared to the rear half of the cells was quantified after 100 or 200 μm of migration through confined or unconfined channels, and the front : rear ratio of active mitochondria was found to be significantly higher in confined cells (Fig. 3F and G), possibly to fuel increased actin polymerization observed at the leading edge (Fig. 3A and B). To further understand the relationship between matrix interaction, migration, and mitochondrial positioning, the speed and front : rear mitochondria ratio of cells migrating in 5, 10, 15 and 20 μm width tracks were compared. Both speed and the amount of active mitochondrial content in the cell front increased with decreasing width microtracks as cells became more confined, exhibiting a linear relationship between degree of confinement and front : rear mitochondria or speed (Fig. 3H). Disrupting activity of mitochondria with the electron transport chain inhibitor, antimycin-A, not only decreased migration speed in confined microtracks (ESI Fig. 4A and B†), but also disrupted active mitochondrial localization to the front of the cell (ESI Fig. 4C†), indicating that localized active mitochondria are required for fast MDA-MB-231 migration in confinement, likely to fuel cytoskeletal rearrangement needed with increased matrix contact.

**Fig. 4:**
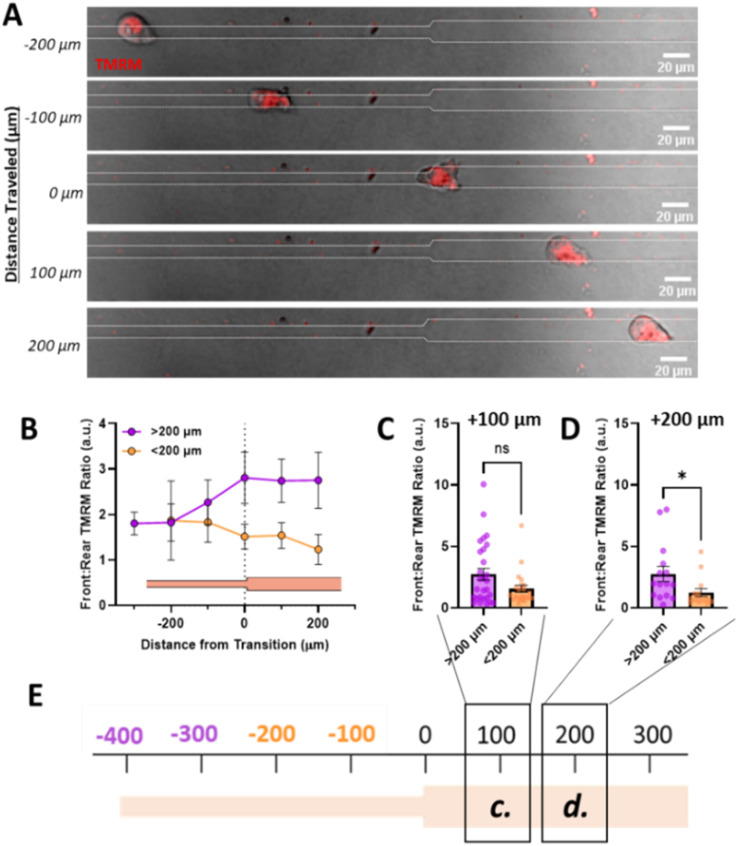
Priming in confinement results in increased mitochondrial localization to the cell front. (A) Representative image of cell moving through temporarily confined channel labelled with TMRM, scale bar = 20 μm; (B) front : rear TMRM signal as a function of position in temporarily confined 7/15 μm where 0 denotes transition, purple indicated cells primed longer than 200 μm and orange indicated cells primed less than 200 μm (*N* = 14, *n* = 25–28); (C and D) front : rear TMRM signal of cells after migrating (C) 100 μm or (D) 200 μm after the transition point in temporarily confined tracks, primed for either <200 μm or >200 μm in the confined region of the track (*N* = 14, *n* = 15–28); (E) schematic of track with transition denoted as 0, with location of measurements in (C) and (D) denoted with boxes/labels. * denotes *p*-value < 0.05.

Since increased front : rear active mitochondrial localization occurs in confinement, to understand how mitochondrial localization relates to memory induced by confinement, we measured mitochondrial front : rear ratio in our temporarily confined microtracks (Fig. 4A). Cells migrating in the confined region of the track maintained high front : rear active mitochondria in the confined portion of the track (Fig. 4B). After transitioning to the unconfined region, front : rear active mitochondria localization in cells that had been primed less than 200 μm in confinement decreased while active mitochondria in cells that were primed greater than 200 μm stayed polarized at the front of the cell (Fig. 4B–E). By 200 μm after the transition point, active front : rear mitochondria were significantly decreased in the group primed for a shorter distance (Fig. 4D). Together, these data indicate that priming in confinement polarizes active mitochondria to the front of the cell, but the extent of priming may determine how long it takes mitochondria to disperse from the front of the cell post-confinement.

### Vinculin controls memory of migrating breast cancer cells

**Fig. 5:**
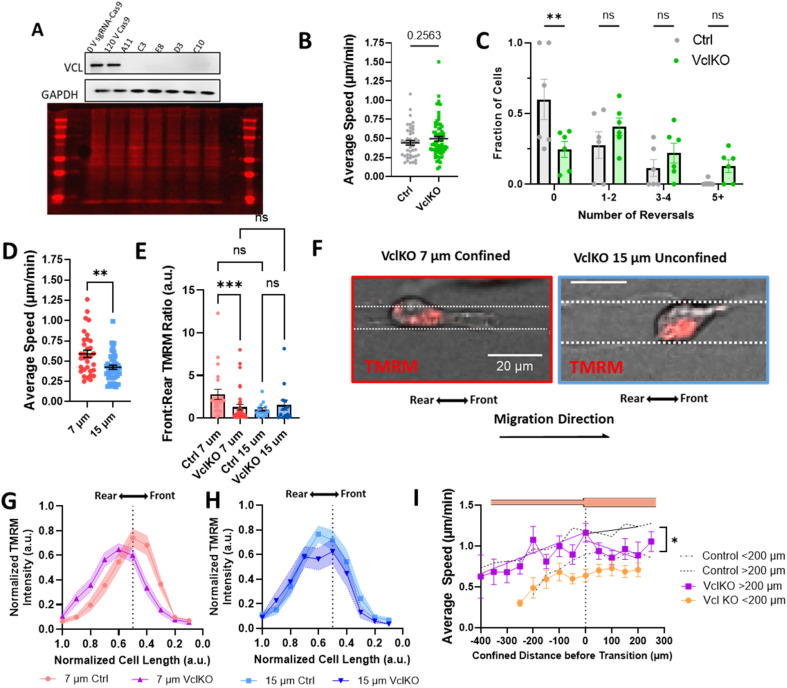
Vinculin is required for priming in confinement. (A) Representative western blot 0 V sgRNA-Cas9 (0 V Ctrl), 120 V Cas9 (Cas9 Ctrl), and VclKO colonies blotted for vinculin (VCL) and GAPDH, and total protein stain; (B) average speed of VclKO cells in 10 μm microtracks (*N* = 6, *n* = 44–66); (C) frequency distribution of number of times a cell reversed direction in 10 μm microtracks (*N* = 6, *n* = 44–66); (D) average speed of VclKO cells in 7 μm or 15 μm microtracks (*N* = 5, *n* = 33–42); (E) front : rear TMRM signal in control or VclKO cells in 7 or 15 μm tracks (*N* = 3, *n* = 14–36); (F) representative images of VclKO cells in confined and unconfined microtracks, scale bar = 20 μm; (G and H) gradient of normalized TMRM signal across length of cell body, normalized to 1 for ctrl or VclKO cells in (G) 7 μm or (H) 15 μm microtracks (*N* = 3, *n* = 14–36 cells); (I) speed of VclKO cells as a function of position in temporarily confined 7/15 μm track, primed for either < or >200 μm (*N* = 6, *n* = 17–19). Black dotted line represents control memory cells from 2B grouped into cells confined < or > 200 μm (*N* = 26, *n* = 53–38). * denotes *p*-value <0.05, **<0.01, Extra Sum-of-Squares F-Test to measure slopes after exit of vinculin KO or control cells in (I).

Since our data indicate that actin and active mitochondria are polarized to the front of the cell in confinement and it has previously been shown that proper function and distribution of mitochondria require intact focal adhesions,^43–46^ we sought to determine whether focal adhesion disruption affected mitochondrial localization during confined migration and priming. We used CRISPR/Cas9 to knock out vinculin expression in MDA-MB-231 cells (Fig. 5A) and measured their migratory ability in 10 μm microtracks. While no difference in speed was observed (Fig. 5B), there was a significant increase in the number of times cells changed directions for vinculin knockout cells (VclKO) (Fig. 5C), agreeing with previous data reporting that vinculin is necessary for persistence in epithelial cells.^31^ Consistent with control cells, VclKO cells in 7 μm confined microtracks exhibit higher speeds than unconfined cells in 15 μm microtracks (Fig. 5D). Interestingly, active mitochondria localization to the front of cells in confinement was significantly decreased for VclKO cells (Fig. 5E–H). Active mitochondria were more localized to the cell center or towards the rear of the cell in confined VclKOs, compared to confined control cells where the majority of active mitochondria were shifted towards the front of the cell body (Fig. 5G). In unconfined tracks, both VclKO and control cells exhibited active mitochondria localized more to the center or rear of the cell body (Fig. 5H).

As depletion of vinculin did not significantly alter migration speed in microtracks but did disrupt mitochondrial localization to the cell front, we next primed VclKO cells in the temporarily confined tracks and measured speed after they exited confinement to determine if vinculin and mitochondrial localization are required for memory. While speed increased in the confined region of the track similar to control cells, once cells had transitioned into the unconfined region, speed quickly decreased and memory of previous confinement was disrupted (Fig. 5I), indicating that vinculin is important to maintaining cell speed after priming.

## Discussion

Cancer cell migration is dictated by cues from the extracellular matrix and surrounding environment.^5,47^ In this study, we aimed to understand not just how cells sense and respond to these cues, but also the longer-term changes associated with cell migration due to signals from the matrix. Dissecting the roles of mechanical cues, cytoskeletal organization, and metabolism in migration requires a highly physiologically relevant system to mimic the architecture of the microenvironment cells encounter *in vivo*. Here, we use a microscale collagen platform fabricated with soft lithography to represent both the geometries and material composition of the native tumor microenvironment to parse the factors driving priming of MDA-MB-231 cells in spatial confinement. Increased matrix contact in confinement correlates with the polarization of key migratory machinery, including actin and mitochondria, which may allow cells to quickly navigate confined spaces. While the absence of vinculin-containing adhesions does not abrogate MDA-MB-231 migration completely, it does disrupt the ability of cells to prime in confined microtracks, preventing mitochondrial localization and maintenance of high speeds.

The physical and mechanistic link between vinculin and mitochondria within the cell is still poorly understood, though it has been reported that mitochondrial activity tightly regulates focal adhesion dynamics.^43,44,48–52^ Disrupting mitochondrial localization and trafficking throughout the cell by knocking out Miro1 in mouse embryonic fibroblasts resulted in significantly decreased paxillin-containing focal adhesion size, and significantly decreased expression of phosphorylated vinculin.^49^ Likewise, the activity of the focal adhesion protein, paxillin, was shown to control mitochondrial fission dynamics through activity of the fission protein, DRP-1, as well as mitochondrial localization within the cell body.^43^ When the focal adhesion protein, integrin-linked kinase (ILK), was knocked down in MDA-MB-231s, mitochondrial localization to the peripheral regions of the cell was disrupted and mitochondria primarily occupied the perinuclear area.^44^ Upon rescue of ILK, mitochondria returned to periphery of the cell. Furthermore, vinculin reportedly tethers mitochondria to generate the energy needed to control focal adhesion size.^53^ Together, these findings point to a link between mitochondria and focal adhesions, where proper mitochondrial distribution and function is required for focal adhesion formation, and proper focal adhesion assembly and protein recruitment influences mitochondrial distribution and fission in the cell.

Since mitochondrial content has been shown to be dependent on proper focal adhesion dynamics,^43,44^ we disrupted focal adhesion formation with the knockout of vinculin to reduce mitochondrial localization to the cell front to determine the effect on confined migration and memory. However, the relationship between mitochondria localization and fast migration is complex. Though mitochondria localization was correlated with fast migration in MDA-MB-231s, when vinculin was removed and mitochondria no longer localized at the front of the cell, we observed no change in speed. It is still unknown whether mitochondria localization to the front of the cell is required for fast migration in confinement, or it is the result of faster migration, as migration machinery also gets polarized to the leading edge of the cell. Previous studies have reported that disrupting mitochondrial motility by knocking down Miro1 results in decreased migration speed and persistence.^25,29^ Deeper parsing of this relationship between mitochondria, vinculin, and migration in future work is critical to fully understand how energetics and cytoskeletal dynamics work together to control cancer cell invasion and migration.

The role of vinculin in migration has been heavily studied in the context of cancer and is known as a critical regulator of force transduction between cells and the surrounding matrix.^31,32,54–58^ Previously, we have seen that cells in confinement increase matrix strains through larger vinculin-containing adhesions.^18^ Additionally, matrix traction forces have recently been shown to play a key role in the polarization and persistent migration of keratocytes,^59^ and tend to be increased at the leading edge of cells.^60,61^ It is possible that in confinement, increased matrix contact encourages mature focal adhesions to grow at the leading edge of cells through increased force transduction, demanding high energy and inducing mitochondria recruitment to the front of the cell. This polarization of cytoskeletal and metabolic structures at the leading edge of cells, where increased actin polymerization aids in the formation of protrusions and adhesions, which drives forward motion, may aid in the maintenance of high speed as cells transition from the highly confined region to the unconfined region in the temporarily confined tracks.

While we uncovered an interesting property of the highly metastatic and representative triple-negative breast cancer cell line, MDA-MB-231, in which they can be primed in a vinculin-dependent manner to increase active mitochondrial polarization in confining regions of increased matrix contact, the mechanism by which vinculin and mitochondria interact to facilitate fast migration is still not fully understood. With this collagen microtrack platform, we are able to mimic the soft, porous matrix encountered by cancer cells *in vivo*. However, because the microtrack platform is a closed system in which cells cannot be removed after confinement, our system limits the technical capability we have to fully explore the mechanism driving the observed phenomenon. For example, we are not able to remove cells from the system to perform bulk assays to assess transcriptional changes, protein regulation, or long-term behavioral effects. Further, because we are tracking single cell migration over long distances, we are currently limited by how much we can capture before the cells undergo division. The doubling rate of MDA-MB-231 cells is ∼25–30 h, and with our current experimental design (ESI Fig. 1B†), with a 6 h incubation and 12–18 h imaging time, we are not able to track single cells for extended times or distances. With current and future work to optimize our model, we hope to overcome this limitation to further investigate the long-term changes on migration due to confinement. While here we have uncovered a phenomenon in which cell priming in confined, collagen matrices may result in relatively short maintenance of memory (<24 h), future work is aimed at determining the total length of this memory, and whether it is a permanent change to the cell, or the effect dissipates over time.

Despite being a low-throughput system, the value of accurately modeling the stiffness ranges and matrix interactions of the native tumor is essential to probing cancer cell behavior. By understanding the nuanced interaction between cancer cells and the matrix, key targets may be exposed for understanding and developing therapeutics to treat metastasis.

## Conclusions

In this study, we observed a novel phenomenon in which migrating MDA-MB-231 cells in soft collagen microtracks can be primed in confinement to change migratory behavior. Consistent with recent findings by other groups suggesting that confinement increases polarization of migratory equipment,^12,13,29^ we observe that the increased matrix contact in confined microtracks correlates with the polarization of key machinery, like actin and active mitochondria, which may allow for fast migration after priming in confinement. Furthermore, the loss of active mitochondrial localization to the front of a migrating cell when the focal adhesion protein, vinculin, is knocked out correlates with a decrease in cell speed after exit from confinement, suggesting a disruption of memory. Though the specific mechanism by which mitochondria and focal adhesion dynamics are linked to drive confined migration are still unknown, this work describes one mechanism by which cellular priming in confinement may increase future migration speed. In future work, we aim to further define the linkage between cytoskeletal dynamics, metabolism, and migration in a larger scale to better understand how cues from the microenvironment regulate breast cancer cell behavior long-term.

## Author contributions

C. A. R. K. and J. A. M. contributed to conceptualization. J. A. M., E. D. F., C. M. L., and A. E. W. contributed to data collection and formal analysis. J. A. M. and C. A. R. K. contributed to writing and editing. C. A. R. K. contributed to funding acquisition, investigation, project administration, data curation, and supervision. J. A. M. contributed to visualization.

## Conflicts of interest

There are no conflicts to declare.

## Supplementary Material

NA-006-D3NA00478C-s001

NA-006-D3NA00478C-s002
