# Supplementary material for: Confinement primes cells for faster migration by polarizing active mitochondria
Source: Nanoscale Adv. 2023 Nov 22;6(1):209–20. doi: 10.1039/d3na00478c (PMC10729874; doi:10.1039/d3na00478c)
Supplement: NA-006-D3NA00478C-s001 [file NA-006-D3NA00478C-s001.pdf]

Electronic Supplementary Material (ESI) for Nanoscale Advances.
This journal is © The Royal Society of Chemistry 2023

Cropped Blot for Figure 5A

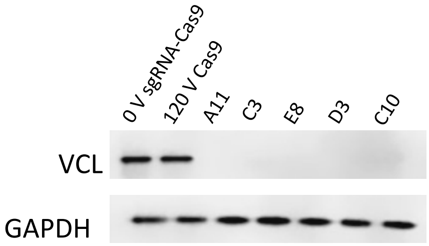

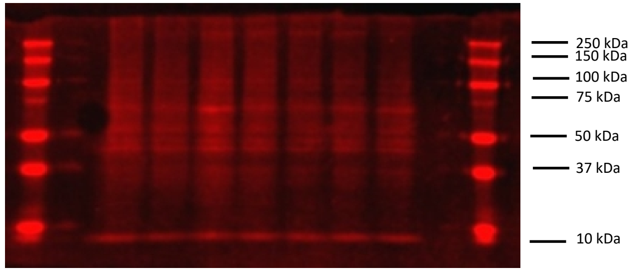

Total protein stain to show loading and ladder.

Uncropped Blots

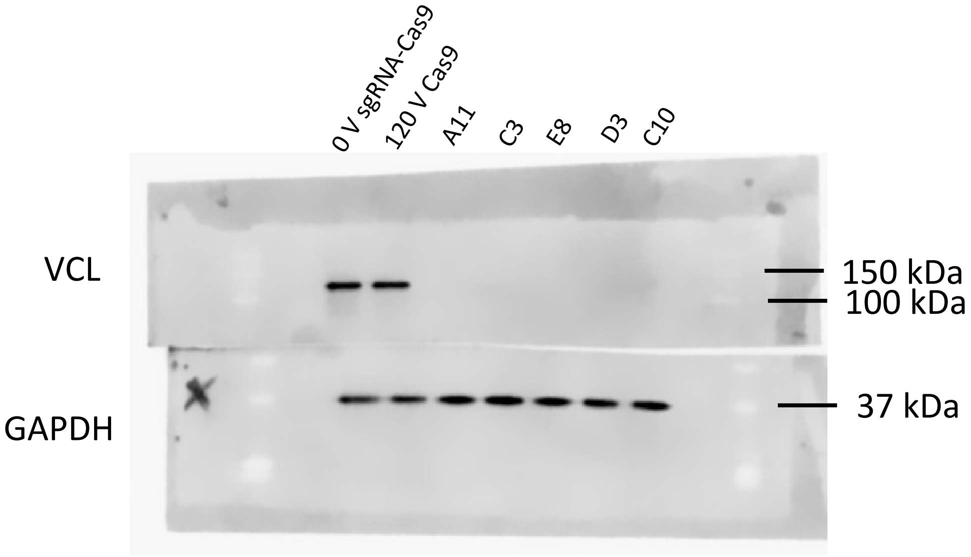

- After transferring protein from gel to PVDF membrane, performed total protein stain and imaged in 700 nm channel.
- After total protein stain, cut membrane between 50 kDa and 75 kDa bands.
- Stained top half of membrane with vinculin and bottom half with GAPDH primary antibodies and then incubated both membranes with IR Dye 800 secondary antibody.
- Images blots in the 800 channel
- Ladder used is from Bio-Rad (Cat #1610375)
