# Supplementary material for: Confinement primes cells for faster migration by polarizing active mitochondria
Source: Nanoscale Adv. 2023 Nov 22;6(1):209–20. doi: 10.1039/d3na00478c (PMC10729874; doi:10.1039/d3na00478c)
Supplement: NA-006-D3NA00478C-s002 [file NA-006-D3NA00478C-s002.pdf]

Electronic Supplementary Material (ESI) for Nanoscale Advances.
This journal is © The Royal Society of Chemistry 2023

Confinement primes cells for faster migration by polarizing active mitochondria

Supplementary Information File

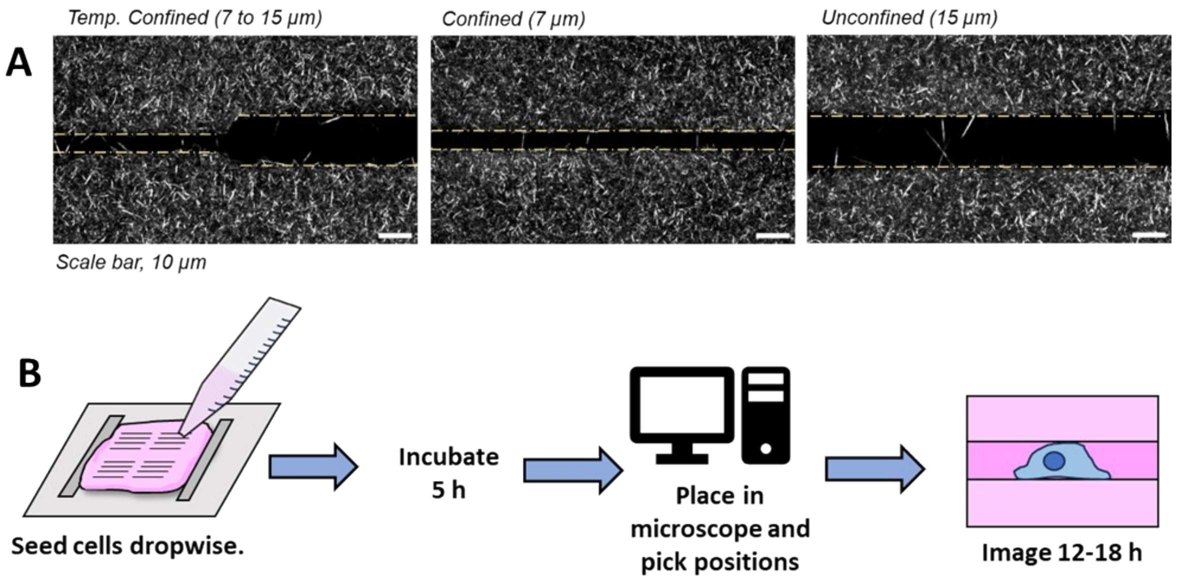

Supplementary Figure 1. Fabrication and use of collagen microtrack platform. A) Representative confocal reflectance images taken of collagen microtrack system, temporarily confined, confined, and unconfined microtracks shown; B) Overview schematic of technique for micromolding collagen and seeding cells. Cells are seeded onto molded collagen after it has been stamped and polymerized, the entire system is capped and placed in a 37°, 5% CO_2 incubator and left for 5 h to allow for cell spreading and attachment. After 5 h, cells are placed in environmental chamber attached to a confocal microscope and allowed to come to temperature while picking positions to image. Cells are then imaged overnight for 12-18 h.

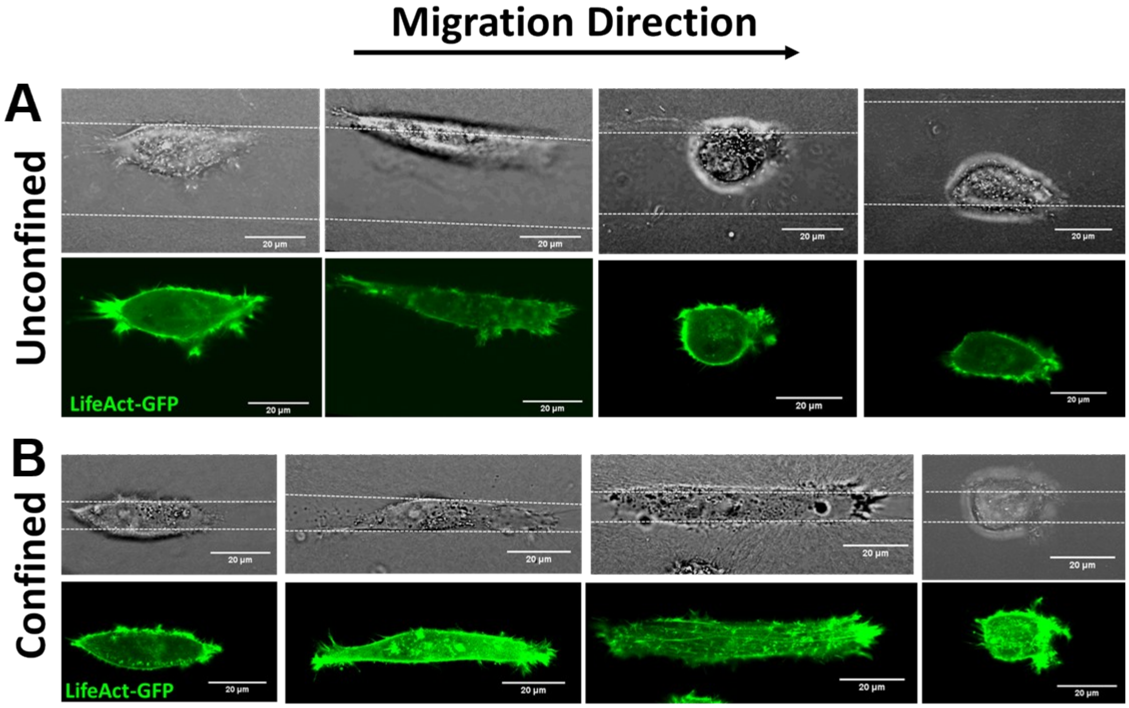

Supplementary Figure 2. Actin organization is cortically located with few stress fibers in microtrack platform. A-B) Representative images taken of LifeAct-GFP-labeled MDA-MB-231 cells in (A) unconfined or (B) confined microtracks, with tracks outlined in white. Scale bar = 20 μm, all cells moving right.

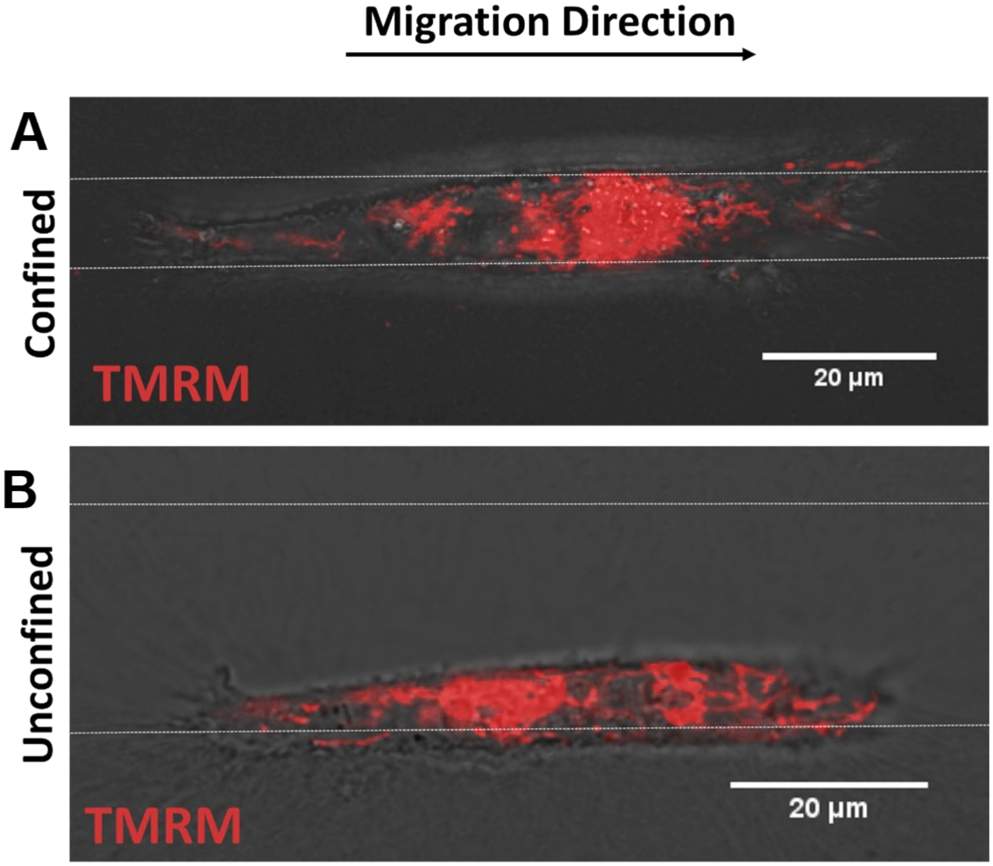

Supplementary Figure 3. Mitochondria localize in the direction of migration in confinement. A-B) Representative images taken of TMRM-labeled MDA-MB-231 cells in (A) confined or (B) unconfined microtracks, with tracks outlined in white. Scale bar = 20 µm, all cells moving right.

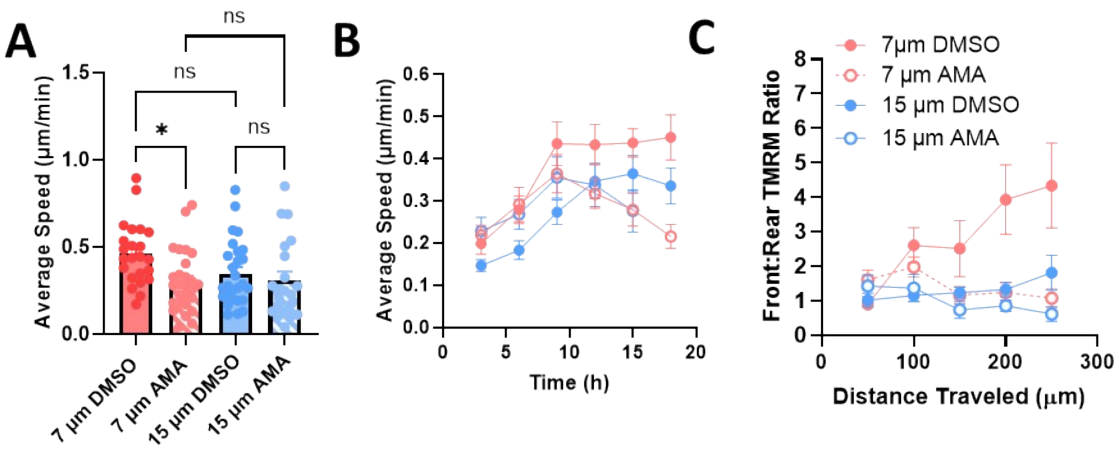

Supplementary Figure 4. Inhibiting of mitochondrial activity correlates with decreased migration velocity in confinement and decreased front:rear active mitochondrial localization. A) Average speed of vehicle control DMSO treated or 500 µm antimycin-A treated MDA-MB-231 cells in 7 or 15 µm tracks averaged 10-18 h after treatment. (N=5-7, n=19-30); B) Speed as a function of time in cells treated with vehicle control or AMA (N=5-7,n=19-30); C) Front:rear TMRM signal in DMSO or AMA treated MDA-MB-231 cells in 7 or 15 µm tracks (N= 5-7, n=19-30).; *denotes p-value <0.05.
